# Elevated blood levels of liver-expressed antimicrobial peptide 2 in patients with insulinoma and its expression in insulinomas

**DOI:** 10.3389/fendo.2025.1685806

**Published:** 2025-12-19

**Authors:** Jia-Lei Zhang, An-Li Tong, Yun-Lu Feng, Da-Chun Zhao, Ling Zhong, Yin-Jie Gao, Yu-Li Song, Tai-Ping Zhang, Ming Li, Yuan-Jia Chen

**Affiliations:** 1 Department of Gastroenterology, Peking Union Medical College Hospital, Chinese Academy of Medical Sciences, Peking Union Medical College, Beijing, China; 2 Department of Endocrinology, Peking Union Medical College Hospital, Chinese Academy of Medical Sciences, Peking Union Medical College, Beijing, China; 3 Department of Pathology, Peking Union Medical College Hospital, Chinese Academy of Medical Sciences, Peking Union Medical College, Beijing, China; 4 Department of Surgery, Peking Union Medical College Hospital, Chinese Academy of Medical Sciences, Peking Union Medical College, Beijing, China

**Keywords:** ghrelin, insulin, insulinoma, LEAP2, obesity, pancreatic neuroendocrine tumors

## Abstract

**Objective:**

Most patients with insulinoma are overweight or obese. Ghrelin levels in insulinoma have been linked to both obesity and hyperinsulinemia. Liver-expressed antimicrobial peptide 2 (LEAP2), a novel hormone and endogenous antagonist of the ghrelin receptor, is associated with obesity; however, its relationship with obesity and hyperinsulinemia in insulinoma, and its expression within tumor tissue, remains unknown.

**Methods:**

Serum LEAP2, insulin, and ghrelin levels were measured by ELISA in patients with insulinoma and in age-, sex-, and BMI-matched controls. LEAP2 expression was examined in insulinoma tissue and paired adjacent pancreatic specimens by immunohistochemical staining.

**Results:**

Serum LEAP2 levels were significantly higher in patients with insulinoma than in controls (P = 0.028) and positively correlated with serum insulin levels (r = 0.408, P < 0.001) while negatively correlated with ghrelin levels (r = −0.551, P < 0.01). In controls, serum LEAP2 levels were significantly associated with BMI (P = 0.047), whereas in patients, the correlation did not reach statistical significance (P = 0.067). Immunostaining showed that LEAP2 peptide was expressed in insulinoma tissue.

**Conclusion:**

Serum LEAP2 levels are elevated in insulinoma and correlate with hyperinsulinemia and BMI. LEAP2 is expressed in insulinoma tissue, suggesting a potential role in the metabolic profile of these patients.

## Introduction

The prevalence of obesity is increasing rapidly worldwide, including in Asia and China (1), and is associated with the development of various tumors and chronic diseases. Over the past four decades, the incidence and prevalence of pancreatic neuroendocrine tumors (PNETs) have also risen substantially (2, 3). PNETs comprise a heterogeneous group of tumors classified as non-functioning and functioning. The classification is clinically important, and functioning PNETs oversecrete unique hormones/peptides and result in specific symptoms or syndromes (4, 5). Insulinoma, a rare but major subtype of functioning PNETs (6, 7), accounts for nearly half of PNETs in China (8). Most patients with insulinoma present with the classic Whipple’s triad due to excessive secretion of insulin or proinsulin by tumor cells (9). Many of these patients are overweight or obese, often as a result of frequent food intake to relieve hunger or prevent hypoglycemic episodes (7, 9, 10). Whether circulating levels of appetite- or obesity-related hormones are altered in insulinoma—particularly among overweight or obese patients—remains unclear. Previous studies reported that 25%–50% of insulinoma patients were overweight or obese, and hypoglycemia markedly enhances hunger sensations, which could partially contribute to increase food intake in these patients (11). Moreover, insulinoma provides a unique natural model for studying the interplay between obesity and endogenous hyperinsulinemia.

Ghrelin, an orexigenic hormone and the endogenous ligand of the growth hormone secretagogue receptor type 1A (GHSR), promotes food intake and plays an important role in regulation of energy balance. In the state of obesity, blood ghrelin levels were typically lower and associated with ghrelin resistance (12–14). Beyond appetite regulation, ghrelin influences the secretion of hormones such as growth hormone, somatostatin, and insulin, modulates glucose homeostasis by inhibiting insulin secretion, and activates the hypothalamic–pituitary–adrenal axis (13, 15–18). Its expression has been revealed in several neuroendocrine tumors (19). Liver-expressed antimicrobial peptide 2 (LEAP2) was recently identified as an endogenous antagonist and inverse agonist of GHSR (20). LEAP2 attenuates ghrelin-induced growth hormone release, suppresses appetite in humans and rodents, and correlates positively with body mass (21–23). Recent evidence suggests that postprandial increases in LEAP2 levels may play a more prominent role in appetite suppression than concurrent decreases in ghrelin (24). LEAP2 is primarily produced by hepatocytes and enterocytes of the small intestine (20, 25, 26), but its physiological and clinical significance remains incompletely understood.

Our previous work demonstrated that circulating ghrelin levels were significantly reduced in patients with insulinoma and associated with obesity (10). However, circulating LEAP2 levels in insulinoma and LEAP2 peptide expression in tumor tissue have not been evaluated.

The present study aimed to determine serum LEAP2 levels in patients with insulinoma, assess their associations with obesity and obesity-related hormones, and examine LEAP2 peptide expression in insulinoma tissue.

## Materials and methods

### Patients, controls, ethics and diagnostic criteria

Between August 2003 and May 2021, we recruited 89 patients with sporadic insulinoma and 40 healthy volunteer controls at our hospital. Controls were matched with patients for age, sex, and body mass index (BMI). Individuals in the control group were excluded if they had endocrine disorders (including endocrine tumors), metabolic syndrome, diabetes, other diseases associated with disordered eating, hepatic or renal dysfunction, or a history of gastric surgery.

The study was conducted in accordance with the Declaration of Helsinki and approved by the Scientific Ethics Committee of our hospital (approval number S-K431). Written informed consent was obtained from all participants prior to inclusion. The dataset for this analysis was accessed on 15 November 2024 for research purposes. No personally identifiable information was accessible to the authors during or after data collection.

Diagnostic criteria for insulinoma followed previous reports (6, 7, 9, 10, 27, 28). Briefly, the diagnosis required (i) symptoms of hypoglycemia; (ii) plasma glucose < 2.8 mmol/L; (iii) hyperinsulinemia, defined as serum insulin ≥ 17.2 μIU/mL during hypoglycemia; and (iv) normalization of blood glucose after tumor resection. Tumor localization was achieved using contrast-enhanced computed tomography, magnetic resonance imaging, and/or endoscopic ultrasound. Pathological diagnoses were confirmed independently by two experienced pathologists. Tumor stage and grade were assessed according to the European Neuroendocrine Tumor Society (ENETS) and World Health Organization (WHO) classifications (29).

Obesity in Chinese adults was defined as BMI≥28 kg/m², and overweight as BMI≥25 but<28 kg/m², according to the *Guidelines for Diagnosis and Treatment of Obesity (in Chinese)* issued by the National Health Commission of the People’s Republic of China in October 2024 (see **Supplementary File 1** for the official PDF version).

### Samples collection and assay

Of the 89 patients, fasting preoperative venous blood samples were obtained from 75 fasting patients with sporadic insulinoma and from 40 fasting matched controls. Postoperative blood samples, collected within the first week after surgery (days 3-7), were available for 4 of the 75 patients. A broad-spectrum protease inhibitor cocktail (cOmplete, Mini, EDTA-free, Roche, Catalog No. 11836170001) was added to all blood samples immediately after collection according to the manufacturer’s instructions. Serum was separated by centrifugation, aliquoted, and stored at −80°C until analysis. Upon thawing on ice, serum samples were centrifuged at 1,500 g for 1 min at 4°C and kept on ice before transfer to assay plates.

Serum LEAP2 concentrations were measured in the 75 patients and 40 controls using a commercially available ELISA kit (EK-075-40, Phoenix Pharmaceutical, USA) following the manufacturer’s instructions, as described by Mani et al. (21), Bhargava et al. (24), and Holm et al. (25). Serum insulin levels were measured concurrently in 74 patients and 28 controls (limited by available serum volume in controls) using an in-house sandwich enzyme-linked immunosorbent assay (ELISA) that was developed and validated by our group for the specific measurement of human insulin, as previously described (27). This assay employs a pair of monoclonal antibodies (McAbs S11 and L11) targeting distinct epitopes on the insulin molecule. The normal cutoff value for insulin was ≤ 17.2 µIU/mL (30). Measurement of serum acylated ghrelin was limited by available serum volume. Acylated ghrelin levels were therefore determined in a subset of 23 patients and 6 controls using an ELISA kit (A05106, Bertin Technologies, France) which was used by van Adrichem et al. (31). All assays were performed in duplicate.

### Detecting LEAP2 expression in positive/negative controls and tumors

Expression of LEAP2 peptide was evaluated by immunohistochemistry (IHC) in a subset of 26 formalin-fixed, paraffin-embedded (FFPE) insulinoma specimens and 13 paired para-tumoral tissues, which were obtained from the surgical resections of the patients. Subsequent to resection, all specimens were processed following standard pathological protocols, including fixation in 10% neutral buffered formalin and paraffin embedding. Following antigen retrieval (microwave heating, 2 min in 0.01 M sodium citrate buffer, pH 6.7, repeated three times), sections were incubated overnight at 4°C with a primary antibody against LEAP2 (Phoenix Pharmaceuticals, H-075-40; dilution 1:100). This antibody was selected because the same Phoenix H-075-40 antiserum was successfully used to detect plasma LEAP2 in rodents, supporting its overall analytical reliability (17). Moreover, our serum measurements used the Phoenix ELISA kit (EK-075-40) for ensuring consistent using Phoenix reagents across both tissue and serum assays. Goat anti-rabbit/mouse IgG conjugated to horseradish peroxidase (HRP) (Beijing Zhongshan Golden Bridge Biotechnology Co., China) served as the secondary antibody, and 3,3′-diaminobenzidine (DAB) was used as the chromogen to visualize positive staining as a brown precipitate. Staining was independently evaluated by two observers blinded to circulating LEAP2 levels. Semiquantitative grading criteria for IHC were consistent with our previous reports (6, 27). Briefly, positive LEAP2 expression in insulinoma was defined as distinct immunoreactivity present in more than 20% of tumor cells (6, 10, 27).

For immunofluorescence staining and co-localization, selected specimens were stained with anther LEAP2 antibody (Abbexa, abx104118, Cambridge, UK; dilution 1:25) and, where indicated, co-stained with an insulin antibody (Santa Cruz Biotechnology, 2D11-H5, sc-8033, Lot #G2904; dilution 1:100). After overnight incubation at 4°C, slides were washed and incubated with species-appropriate secondary antibodies: Goat anti-rabbit IgG H&L conjugated with Alexa Fluor^®^ 488 (Abcam, ab150077, Cambridge, UK; 1:100) for LEAP2, and Goat anti-mouse IgG conjugated with Alexa Fluor^®^ 594 (Proteintech, SA00013-3; 1:500) for insulin. Incubation was carried out for 30 min at 37°C in the dark. Nuclei were counterstained with DAPI (1 μg/mL), and slides were mounted with antifade medium.

Two LEAP2 antibodies (Abbexa abx104118 and Phoenix H-075-40) were used in immunostaining on positive control tissues. Two human liver specimens and one jejunum specimen served as positive controls, whereas interstitial cells within liver tissue were used as internal negative controls.

### Statistical analysis

All statistical analyses were performed using SPSS software, version 26.0 (IBM Corp., Armonk, NY, USA). The Shapiro–Wilk test was used to assess the normality of data distributions. Continuous variables with a normal distribution were compared between two groups using the Student’s *t*-test, whereas non-normally distributed variables or variables without homogeneity of variance were compared using the Mann–Whitney *U* test. Categorical variables were compared using the χ^2^ test. Paired *t*-tests were applied to evaluate differences in pre- and postoperative LEAP2 levels. Correlation analyses were performed using Pearson’s correlation coefficient for normally distributed variables and Spearman’s rank correlation coefficient for non-normally distributed variables. Multiple linear regression analysis was conducted to examine the influence of multiple variables on serum LEAP2 levels. All tests were two-tailed, and a *P*-value ≤ 0.05 was considered statistically significant.

## Results

### Clinicopathological characteristics

All 89 sporadic insulinomas were well differentiated; 66% were classified as G1, and the remaining as G2. Among the 49 patients with follow-up data, 45 (92%) were alive without disease, whereas 4 were alive with metastases or had died.

The clinicopathological characteristics of the 75 patients whose serum LEAP2 levels were measured are summarized in **Table 1**, along with their correlations with LEAP2 concentrations. The 40 fasting controls were matched to patients by age, sex, and BMI. Obesity was present in 48% of patients and 43% of controls. The mean ages of patients and controls were 49 and 47 years, respectively. No significant differences in BMI, age, or sex distribution were observed between the two groups.

**Table 1:** Clinicopathological features and correlation of serum levels of LEAP2 with clinicopathological characteristics.

Clinical features	n	LEAP2 levels ng/ml median (range)	n	p value
Gender, n (%)	n=75		75	
Male	31 (41.3)	19.2 (9.95–70.66)	31	0.228
Female	44 (58.7)	24.5 (2.02–79.1)	44	
Age(years), mean ± SD	n=75,49.1 ± 13.5		75	
Body mass index (BMI)	n=75,28.3 ± 5.0		75	
BMI < 28, n (%)	39 (52.0)	20.99 (2.02–79.1)	39	0.10
BMI ≥ 28, n (%)	36 (48.0)	24.66 (13.08–70.66)	36	
Tumor location, n (%)	n=74		71	
Pancreatic head or neck	35 (47.3)	19.45 (8.32–58.39)	35	0.414
Pancreatic body or tail	36 (48.6)	24.50 (2.02–79.10)	36	
Multiple location	3 (4.1)			
Tumor size, n (%)	n=72		72	
<2.5 cm	69 (95.8)	21.38 (2.02–79.10)	69	0.466
≥2.5 cm	3 (4.2)	29.42 (17.29–44.35)	3	
Metastasis, n (%)	n=74		74	
No	71 (95.9)	21.56 (2.02–79.10)	71	0.241
Yes	3 (4.1)	36.69 (16.34–70.66)	3	
Ki–67, n (%)	n=58		58	
≤2%	40 (69.0)	18.56(2.02–79.10)	40	0.724
>2%	18 (31.0)	21.28(7.18–70.66)	18	
Grade, n (%)	n=59		59	
1	36 (61.0)	20.94 (2.02–79.10)	36	0.804
2	23 (39.0)	21.56 (7.18–70.66)	23	
Stage, n (%)	n=73		73	
I	62 (84.9)	22.58 (2.02–79.10)	62	0.22
II	8 (11.0)	17.39 (10.75–44.35)	8	
III+IV	3 (4.1)	36.69 (16.34–70.66)	3	
Follow–up information				
Available	49 (65.3)			
Disease–free survival, n (%)	45 (91.8)	19.06(2.02–55.25)	46	0.174
Survival with tumor or death, n(%)	4 (8.2)	34.51(10.32–70.66	4	

BMI, body mass index (obesity defined as BMI ≥28 kg/m² per Chinese criteria).

### Fasting serum levels of insulin, LEAP2, and ghrelin in patients and controls

Fasting serum insulin concentrations were significantly higher in 74 patients with insulinoma than in 28 controls (median 18.9 μIU/mL, range 2.8-300 μIU/mL *vs*. median 3.9 μIU/mL, range 2.2-10.7 μIU/mL, *P <*0.0001; **Supplementary Figure 1**).

**Figure 1:**
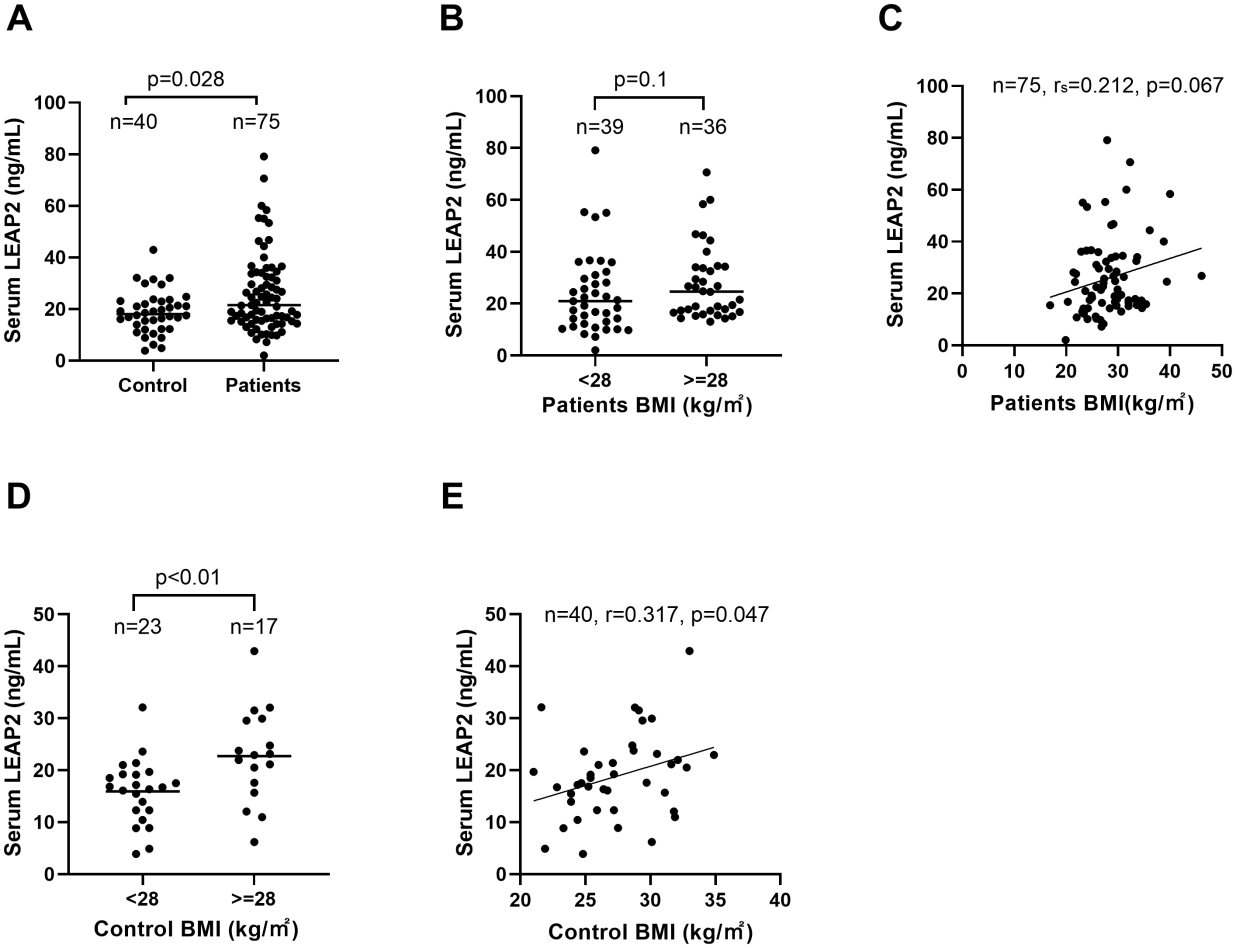
Fasting serum levels of LEAP2 in patients and control group and the relationship between serum LEAP2 levels and obesity. **(A)** Serum levels of LEAP2 in patients with insulinoma were significantly higher than those in control groups (P = 0.028). **(B)** Serum LEAP2 levels in obese patients were not significantly higher than those in non-obese patients (P= 0.10). **(C)** Serum levels of LEAP2 in patients showed a trend toward correlation with obesity (P = 0.067). **(D, E)** Serum levels of LEAP2 in control group significantly correlated with obesity. Each dot represents the LEAP2 level of an individual patient or control.

Median serum LEAP2 levels were also significantly elevated in 75 patients compared with controls (21.6 ng/mL, range 2.0–79.1 *vs*. 18.0 ng/mL, range 3.9–42.9 ng/mL; *P* = 0.028; **Figure 1A**; **Supplementary Table 1**). In patients, there was a trend toward higher LEAP2 levels in obese individuals than those in non-obese individuals (median 24.7 ng/mL, range 13.08-70.66 ng/mL *vs*. median 21.0 ng/mL, range 2.02 - 79.1, *P* = 0.10; **Figure 1B**) and showed a trend toward correlation with BMI (*P* = 0.067; **Figure 1C**). In contrast, obese controls had significantly higher LEAP2 levels than non-obese controls (median 22.9 ng/mL, range 6.18-42.93 ng/mL *vs*. median 16.7 ng/mL, range 3.89-32.11 ng/mL; *P* < 0.01; **Figure 1D**), and LEAP2 concentrations in controls were positively correlated with BMI (*r* = 0.317, *P* = 0.047; **Figure 1E**). These findings confirm a strong association between LEAP2 and obesity in healthy controls. In patients with insulinoma, however, this association is less pronounced and did not reach statistical significance.

In patients, fasting serum insulin levels were significantly associated with obesity. Median BMI was higher in patients with hyperinsulinemia than in those with normal insulin levels (29.4, range 22.0-46.1 *vs*. 25.8, range 16.9-32.0 kg/m²; *P* < 0.001; **Supplementary Figure 2A**). Serum insulin levels correlated positively with BMI in patients (*r* = 0.514, *P* < 0.0001; **Supplementary Figure 2B**) but not in controls (*r* = 0.240, *P* = 0.228; **Supplementary Figure 2C**).

**Figure 2:**
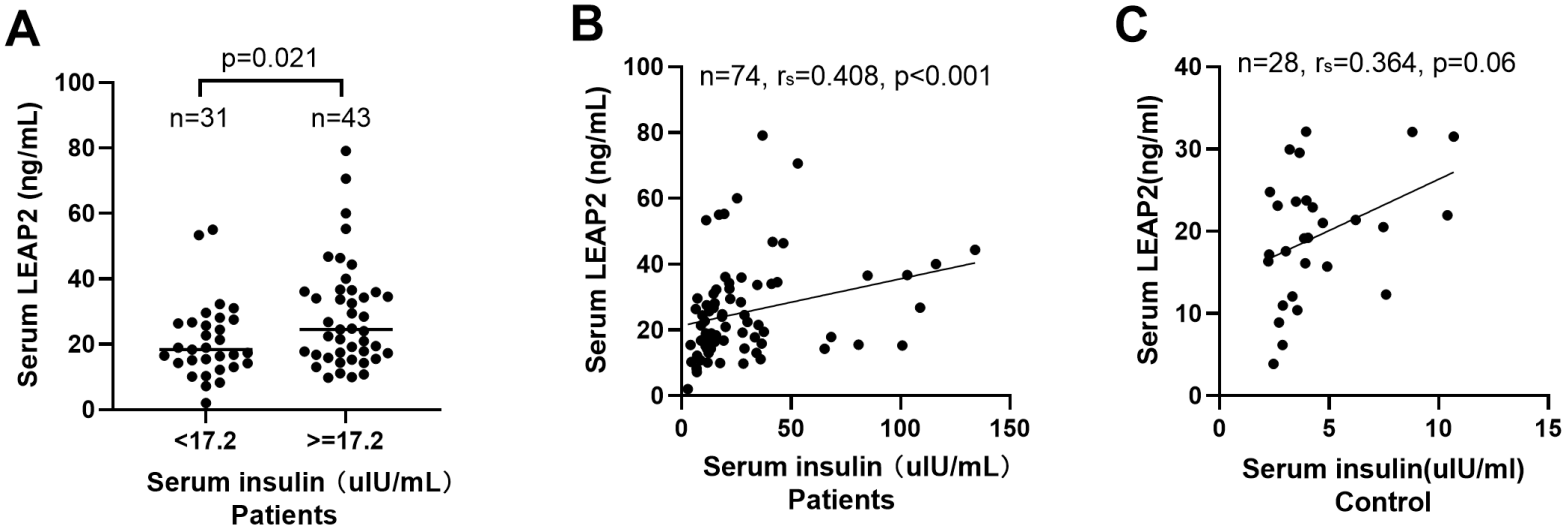
Relationship between fasting serum levels of LEAP2 and insulin. **(A)** LEAP2 levels in patients with hyperinsulinemia (> 17.2 µIU/ml) were significantly higher than those in patients with normal insulin levels (P = 0.021). **(B)** Serum LEAP2 levels positively correlated with insulin levels in patients with insulinoma. **(C)** Serum levels of LEAP2 showed a trend toward correlation with insulin levels but did not reach significance (P=0.06) in controls.

Patients with hyperinsulinemia had significantly higher serum LEAP2 levels than those without hyperinsulinemia (median 24.6 ng/mL, range 9.95-79.1 ng/mL *vs*. 18.4 ng/mL, range 2.02-16.34 ng/mL; *P* = 0.021; **Figure 2A**). In patients, LEAP2 concentrations correlated positively with insulin levels (*r* = 0.408, *P* < 0.001; **Figure 2B**), whereas in controls, the correlation was weaker and not statistically significant (*r* = 0.364, *P* = 0.06; **Figure 2C**). Multivariate analysis identified serum insulin levels as an independent factor associated with LEAP2 concentrations (**Supplementary Table 2**).

Fasting serum ghrelin levels were significantly lower in 23 patients with insulinoma than in controls (median 4.2, range 1.4-17.9 pg/mL *vs*. 14.8, range 4.8-44.3 pg/mL; *P* < 0.01; **Supplementary Figure 3A**), consistent with our previous findings (10). LEAP2 concentrations in patients negatively correlated with ghrelin levels (*r* = −0.551, *P* < 0.01; **Supplementary Figure 3B**).

Paired pre- and postoperative LEAP2 measurements were available for four patients. LEAP2 levels decreased by 29%, 51%, and 54% in three patients postoperatively, but increased slightly (17%) in one patient. Overall, the postoperative decrease was not statistically significant (*t* = 1.791, *P* = 0.171; **Supplementary Figure 4**).

### Expression of LEAP2 peptide in insulinoma and control tissues

Strong LEAP2 peptide expression was observed in positive control tissues using two antibodies. Hepatocytes showed distinct cytoplasmic staining with using either the Phoenix (H-075-40) or Abbexa (abx104118) antibody; no expression of LEAP2 was found in interstitial cells within liver tissue, which could be served as internal negative controls (**Supplementary Figures 5A, 5C**). Jejunal epithelial cells displayed strong LEAP2 staining with both antibodies, confirming their expected expression pattern (**Supplementary Figures 5B, D**). These results indicated the reliability of both antibodies for subsequent tumor analyses.

**Figure 3:**
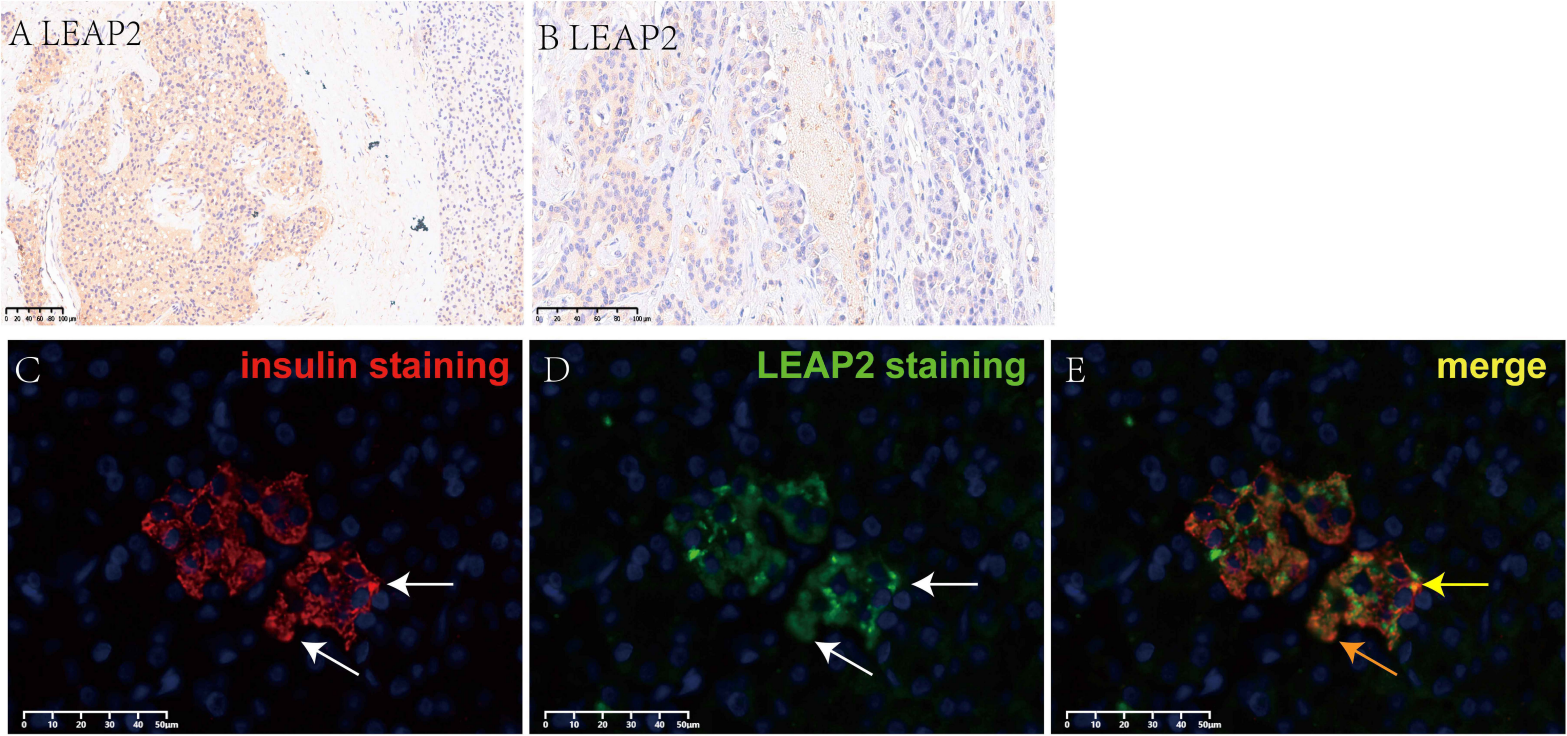
Immunohistochemical staining of LEAP2 and immunofluorescence co-staining of LEAP2 and insulin in insulinoma. **(A)** IHC staining of LEAP2 in an insulinoma, relatively strong expression of LEAP2 in tumor (left part) and no expression of LEAP2 in para-tumoral pancreatic tissue (right part). **(B)** Relatively weak expression of LEAP2 in anther insulinoma (left part) and no expression of LEAP2 in para-tumoral pancreatic tissue (right part). **(C)** Insulin staining (red) using an anti-insulin antibody (Santa Cruz Biotechnology, 2D11-H5, sc-8033; dilution 1:100). **(D)** LEAP2 staining (green) using an anti-LEAP2 antibody (Abbexa, abx104118; dilution 1:25). **(E)** Merged image showing co-localization of LEAP2 with insulin in tumor cells. Nuclei were counterstained with DAPI (blue).

LEAP2 expression was common in insulinomas (**Figures 3A, B**), being detected in 23 of 26 cases (89%), compared with only 3 of 13 paired para-tumoral tissues (23%; *P* < 0.0001). Using another antibody, we performed colocalization for LEAP2 and insulin via immunofluorescence staining, and we found a strong positive signal of LEAP2 within the insulinoma cells (**Figures 3C–E**), confirming the expression of LEAP2 peptide in tumor cells.

### Correlating blood levels of LEAP2 and LEAP2 expression in tumor with clinicopathological features

In patients with insulinoma, serum LEAP2 concentrations were not significantly associated with clinicopathological features (**Table 1**, right panel). Patients with a larger tumor burden—defined as a primary tumor size ≥ 2.5 cm, the presence of multiple pancreatic tumors, or tumor metastases—showed a trend toward higher serum LEAP2 levels compared with those with a lower tumor burden (median 30.9 ng/mL, range 2.02-79.1 ng/mL *vs*. 20.2 ng/mL, range 14.28-70.66 ng/mL; *P* = 0.118; **Supplementary Figure 6**). LEAP2 expression in insulinoma tissues was not significantly correlated with clinicopathological characteristics (**Supplementary Table 3**).

## Discussion

This study was conducted to investigate the role of liver-enriched antimicrobial peptide-2 (LEAP2) in insulinoma, a unique human model of endogenous hyperinsulinemia and obesity. We focused on whether circulating LEAP2 levels are altered in patients, how they relate to insulin and ghrelin, and whether LEAP2 is expressed in tumor tissues.

There are several reasons to explore LEAP2 in this context. First, patients with insulinoma frequently develop obesity due to recurrent hypoglycemia and compensatory food intake, making them a natural model to study obesity-related hormones (9, 10, 32–34). Second, our previous study demonstrated that circulating ghrelin levels are significantly decreased in insulinoma and strongly negatively correlated with hyperinsulinemia (10). Whether LEAP2 shows similar associations was unknown. Third, the expression of LEAP2 in insulinoma tissue had not previously been identified.

The present study provides two novel findings: (i) serum LEAP2 concentrations are significantly elevated in patients with insulinoma and correlate with insulin levels, and (ii) LEAP2 peptide is extensively expressed in insulinoma tissues.

**Figure 4:**
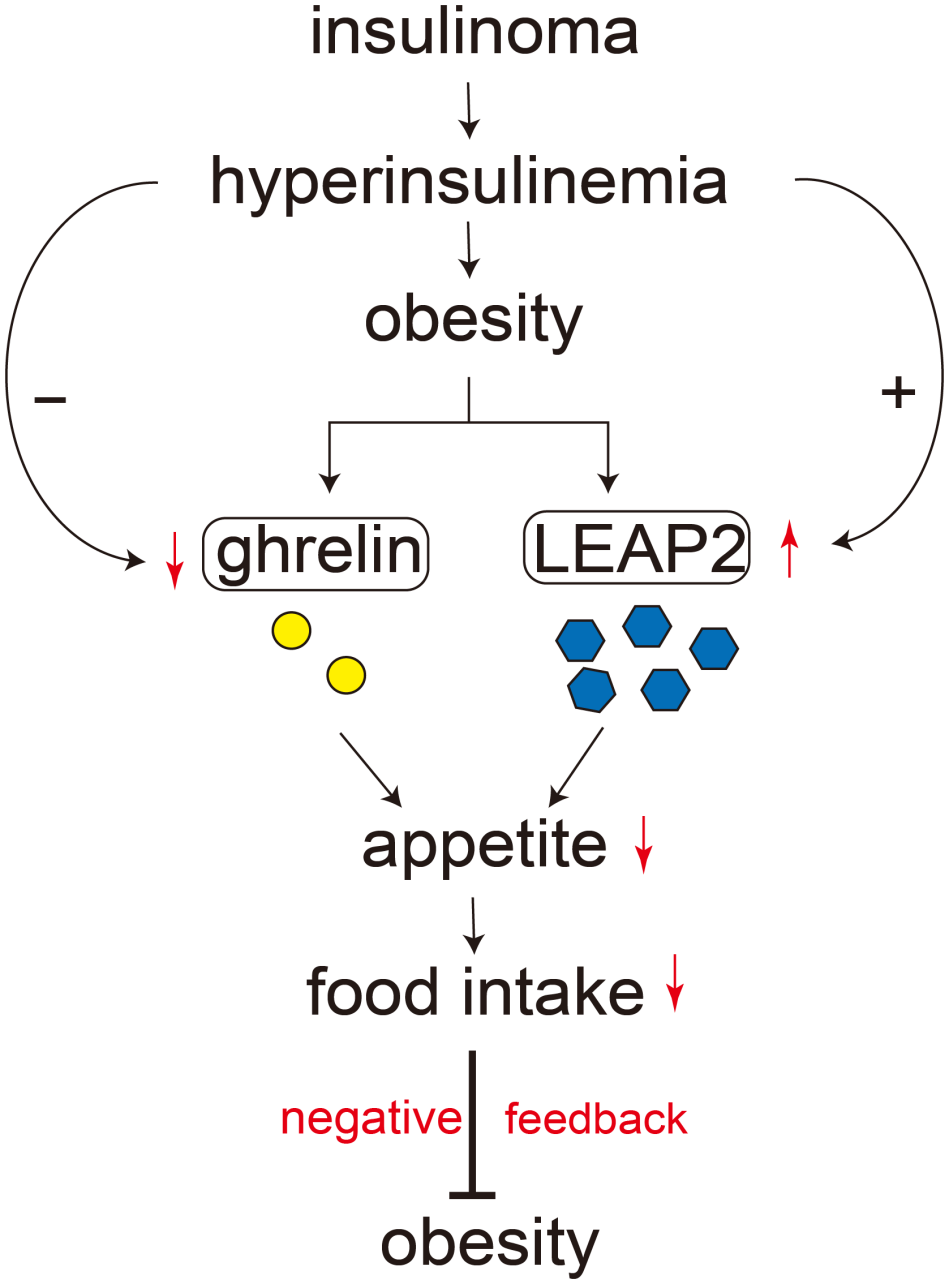
Schematic illustration of the potential interplay between LEAP2, ghrelin, insulin, and obesity. Chronic hyperinsulinemia may contribute to increased circulating LEAP2 and suppressed ghrelin levels, which could together reduce appetite and food intake. This proposed feedback model summarizes possible mechanisms supported by our findings.

Uncontrolled insulin hypersecretion in insulinoma is an autonomous process, independent of glucose or other regulatory hormones. We observed a positive correlation between serum LEAP2 and insulin concentrations, and multiple regression analysis confirmed insulin as an independent determinant of LEAP2 levels (**Supplementary Table 2**). Given that hepatocytes commonly express insulin receptors, we hypothesize that endogenous hyperinsulinemia may partly drive hepatic secretion of LEAP2 in patients with insulinoma (**Figure 4**). Most recent research showed that action of insulin was required for upregulation of LEAP2 but exogenous insulin was not sufficient to increase blood LEAP2 levels (35). In our present study, endogenous hyperinsulinemia in patients with insulinoma was positively correlated with circulating LEAP2 levels, supporting Johansen’s finding (35).

LEAP2 expression was identified in 89% of insulinomas, and patients with a larger tumor burden tended to have higher serum LEAP2 levels (**Supplementary Figure 6**). Moreover, LEAP2 levels declined in three of four cases after resection, suggesting that tumor removal may reduce circulating LEAP2. Taken together, these findings suggested that tumor-derived LEAP2 might contribute to elevated circulating levels of LEAP2.

Our results also highlight the relationship between LEAP2 and obesity. Serum LEAP2 levels were significantly higher in obese than in non-obese controls (**Figure 1D**), consistent with previous studies (25, 26). Recent studies showed that LEAP2 levels were increased postprandially and both peripheral and central administration of LEAP2 suppressed food intake (21, 36). Interestingly, several patients in our cohort exhibited severe obesity; one patient’s BMI value was up to 46. In such obese cases, markedly increased blood levels of LEAP2 and reduced levels of ghrelin might act in concert to inhibit appetite and prevent potentially fatal risks. These findings likely reflected a negative feedback regulation of appetite by LEAP2 in patients with insulinoma (**Figure 4**). However, this relationship could be more complex than we expected and further study including more cases of insulinoma would be needed.

Ghrelin regulates the secretion of multiple hormones (13, 37, 38), including insulin (13, 37–40). In the present study, serum LEAP2 levels were elevated and negatively correlated with blood ghrelin levels, consistent with our previous report showing significantly reduced ghrelin in patients with insulinoma (10). These findings suggest that a delicate balance between ghrelin and LEAP2 may be maintained in patients with insulinoma.

Severe obesity is often accompanied by impaired ghrelin signaling, i.e., ghrelin resistance (24, 25), and a recent study showed that obesity could increase the expression of LEAP2 mRNA in mice liver and induce both ghrelin and LEAP2 resistance in mice (41). Bhargava et al. further demonstrated that postprandial rises in LEAP2 may play a more important role in appetite suppression than decreases in ghrelin (24). The interactive relationship between ghrelin and LEAP2 may regulate pathophysiological status in insulinoma. Together with previous evidence, our findings support the concept that LEAP2 represents a promising therapeutic target for obesity (21, 41, 42).

We also confirmed robust LEAP2 expression in insulinoma specimens using two independent antibodies, with appropriate positive and negative controls to ensure methodological reliability. Our earlier work demonstrated that the LEAP2/ghrelin receptor is expressed in more than half of insulinomas (10), consistent with studies by Ekeblad (43) and Volante (44). The co-expression of LEAP2 and its receptor suggests that LEAP2 may exert autocrine and/or paracrine effects in insulinoma, similar to ghrelin (10, 45). Given that ghrelin inhibits insulin release (39, 40, 46), it will be important to determine whether LEAP2 has the opposite effect, potentially enhancing insulin secretion or promoting tumor proliferation. Indeed, recent studies have shown that LEAP2 can modulate islet hormone secretion and, in some contexts, promote insulin release (47, 48). Such studies may yield novel insights into insulinoma pathogenesis.

This study has several limitations. It was retrospective in design, and functional assays to directly assess the effects of LEAP2 on insulin secretion were not performed. Postoperative serum samples were limited to only four patients, restricting our ability to fully assess dynamic changes. Strengths of this study include the concomitant measurement of three appetite- and obesity-related hormones, the use of validated antibodies to confirm LEAP2 expression, and a relatively large patient cohort for a rare tumor, which enabled meaningful analyses in this distinctive disease model.

In conclusion, serum LEAP2 levels are significantly elevated in patients with insulinoma, closely associated with hyperinsulinemia, and possibly linked to obesity. Extensive tumor expression of LEAP2 indicates both hepatic and tumor-derived contributions. Leveraging the unique “insulinoma model” provides valuable insights into the complex interactions between hormones, obesity, and tumor biology.

## Data Availability

The original contributions presented in the study are included in the article/**Supplementary Material**. Further inquiries can be directed to the corresponding authors.

## Supplementary material

Supplementary Figure 1 Hyperinsulinemia in patients with insulinoma. Serum levels of insulin in insulinoma patients were significantly higher than that in controls (P< 0.0001). Each dot represents an individual value of insulin in patient or control group.

Supplementary Figure 2 Serum levels of insulin and obesity. **(A)**, BMI values in patients with hyperinsulinemia (> 17.2 uIU/ml) were significantly higher than those in patients with normal insulin levels (P < 0.001); **(B, C)**, Serum insulin levels were significantly correlated with BMI in patients (r= 0.514, P < 0.0001) but not in controls (r = 0.20, P = 0.301). Each dot represents an individual value of BMI.

Supplementary Figure 3 Serum levels of ghrelin in patients and controls. **(A)**, Decreased serum levels of ghrelin in patients with insulinoma (P < 0.01). **(B)**, Serum levels of LEAP2 in patients with insulinoma were negatively correlated with ghrelin levels (r= -0.551, P < 0.01). Each dot represents an individual value of ghrelin for each subject; comparisons between groups were performed using the Mann–Whitney U test.

Supplementary Figure 4 Serum LEAP2 Levels in preoperative and postoperative insulinoma patients. Each dot represents an individual value of serum LEAP2 for each patient (n = 4); pre- and postoperative levels were compared using a paired t-test.

Supplementary Figure 5 Immunohistochemical validation of two LEAP2 antibodies in human control tissues. LEAP2 staining is shown using the Phoenix **(A, B)** and Abbexa **(C, D)** antibodies. Strong LEAP2 expression was observed in hepatocytes and jejunal epithelium **(A–D)**, whereas interstitial cells within liver tissue were unstained and served as internal negative controls **(A, C)**. These findings confirm specific and consistent LEAP2 immunoreactivity across two independent antibodies.

Supplementary Figure 6 Higher serum LEAP2 levels in patients with larger tumor burden. Each dot represents an individual level of LEAP2.
