# Supplementary figures and images for: Elevated blood levels of liver-expressed antimicrobial peptide 2 in patients with insulinoma and its expression in insulinomas
Source: Front Endocrinol (Lausanne). 2025 Dec 19;16:1685806. doi: 10.3389/fendo.2025.1685806 (PMC12757234; doi:10.3389/fendo.2025.1685806)

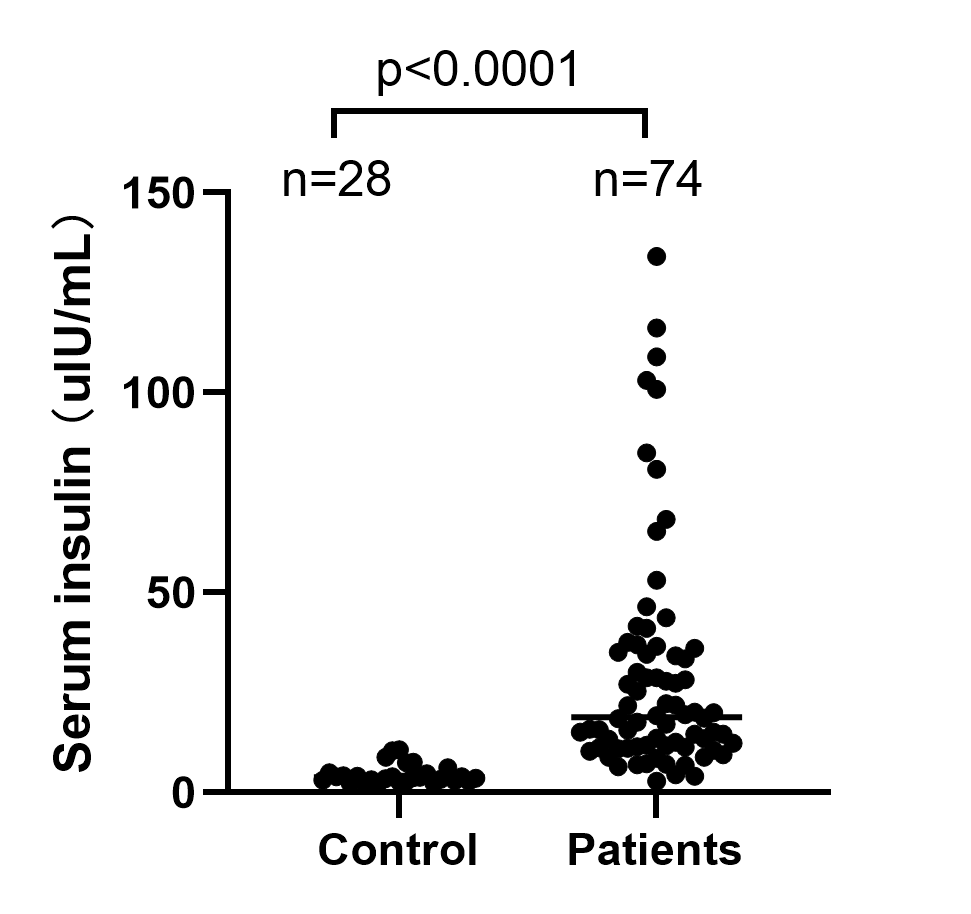

Supplement: Supplementary Figure 1 — Hyperinsulinemia in patients with insulinoma. Serum levels of insulin in insulinoma patients were significantly higher than that in controls (P< 0.0001). Each dot represents an individual value of insulin in patient or control group. [file Image1.tif]

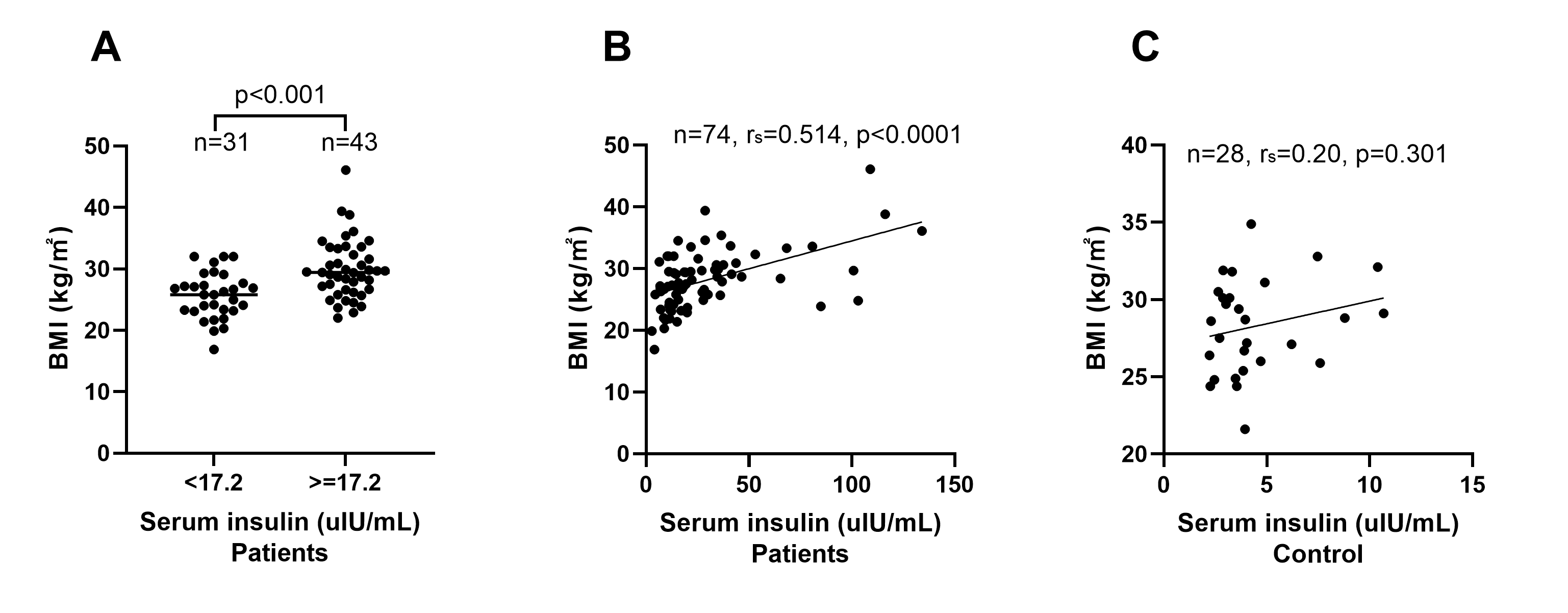

Supplement: Supplementary Figure 2 — Serum levels of insulin and obesity. (A), BMI values in patients with hyperinsulinemia (> 17.2 uIU/ml) were significantly higher than those in patients with normal insulin levels (P < 0.001); (B, C), Serum insulin levels were significantly correlated with BMI in patients (r= 0.514, P < 0.0001) but not in controls (r = 0.20, P = 0.301). Each dot represents an individual value of BMI. [file Image2.tif]

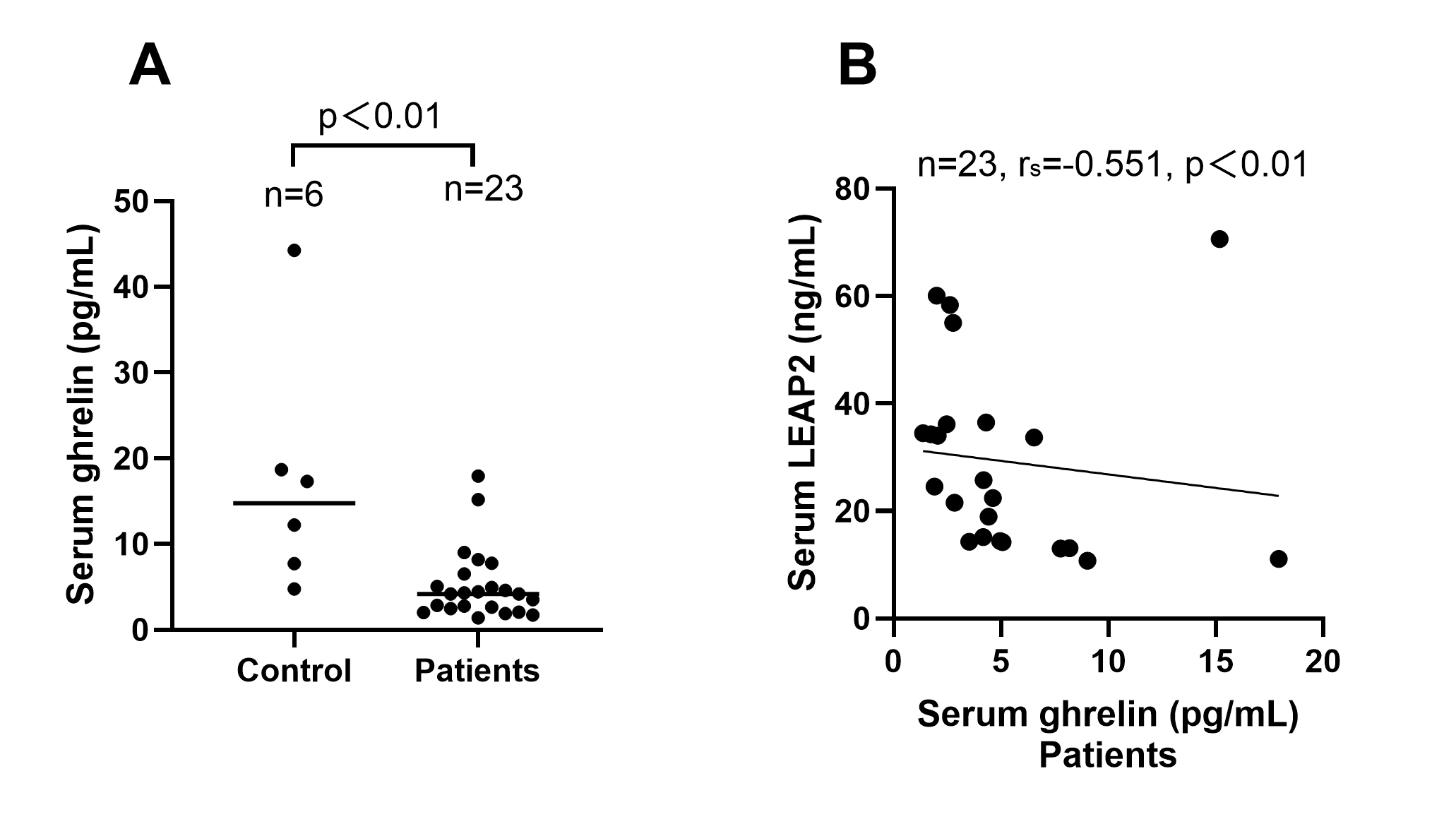

Supplement: Supplementary Figure 3 — Serum levels of ghrelin in patients and controls. (A), Decreased serum levels of ghrelin in patients with insulinoma (P < 0.01). (B), Serum levels of LEAP2 in patients with insulinoma were negatively correlated with ghrelin levels (r= -0.551, P < 0.01). Each dot represents an individual value of ghrelin for each subject; comparisons between groups were performed using the Mann–Whitney U test. [file Image3.tif]

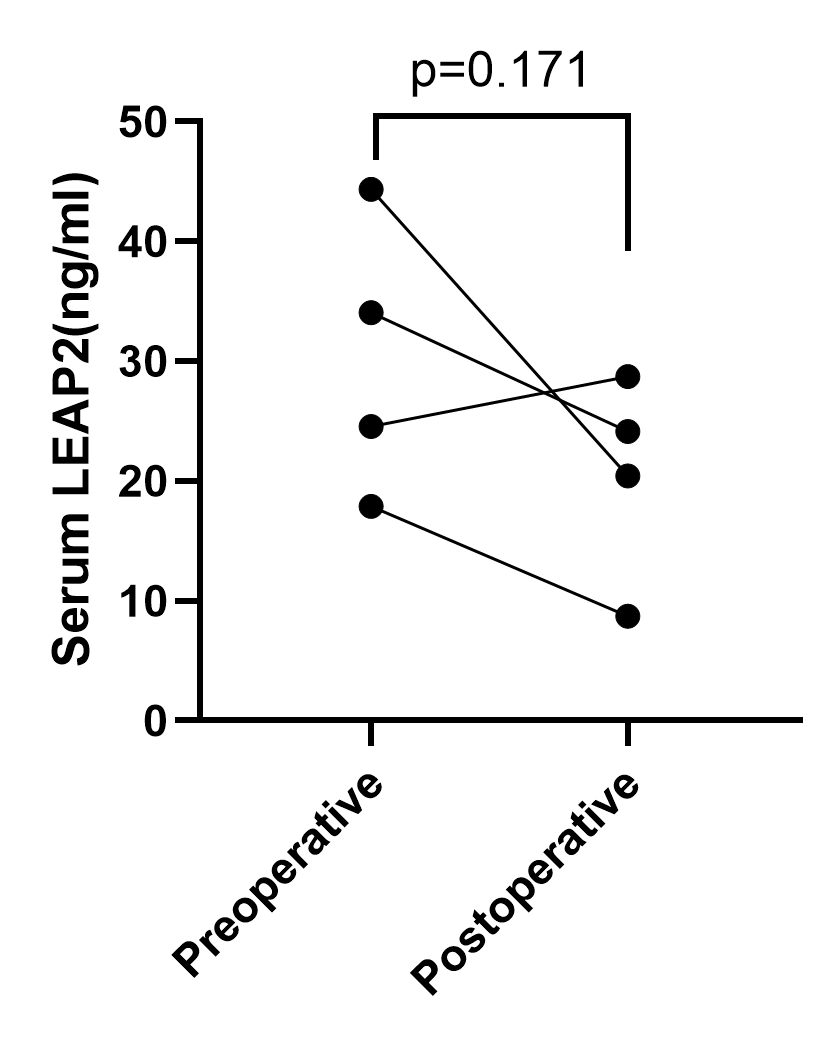

Supplement: Supplementary Figure 4 — Serum LEAP2 Levels in preoperative and postoperative insulinoma patients. Each dot represents an individual value of serum LEAP2 for each patient (n = 4); pre- and postoperative levels were compared using a paired t-test. [file Image4.tif]

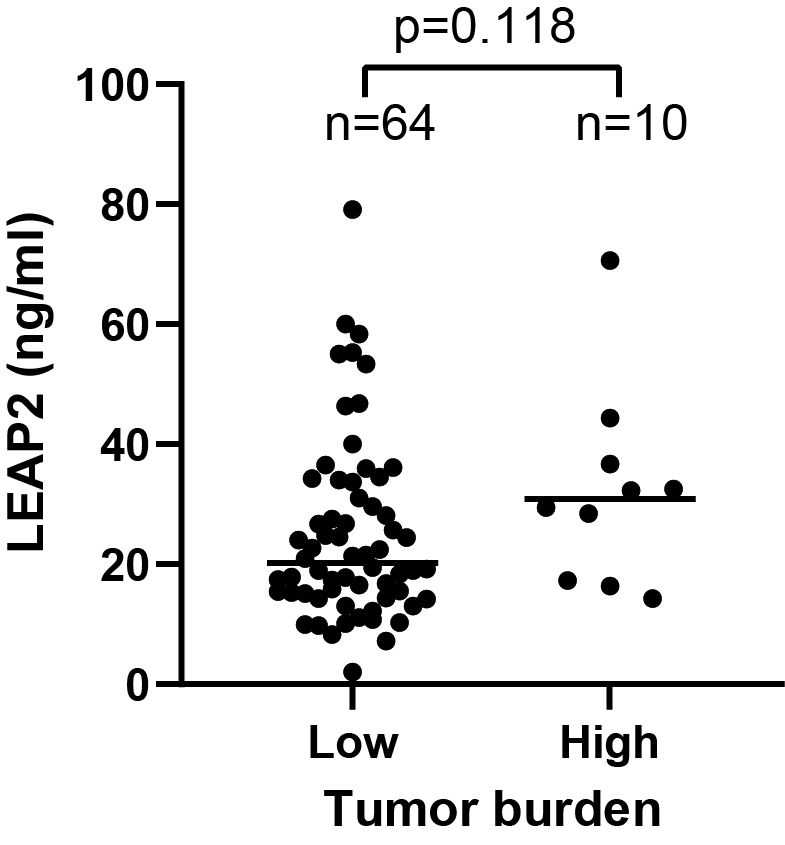

Supplement: Supplementary Figure 5 — Immunohistochemical validation of two LEAP2 antibodies in human control tissues. LEAP2 staining is shown using the Phoenix (A, B) and Abbexa (C, D) antibodies. Strong LEAP2 expression was observed in hepatocytes and jejunal epithelium (A–D), whereas interstitial cells within liver tissue were unstained and served as internal negative controls (A, C). These findings confirm specific and consistent LEAP2 immunoreactivity across two independent antibodies. [file Image5.tif]

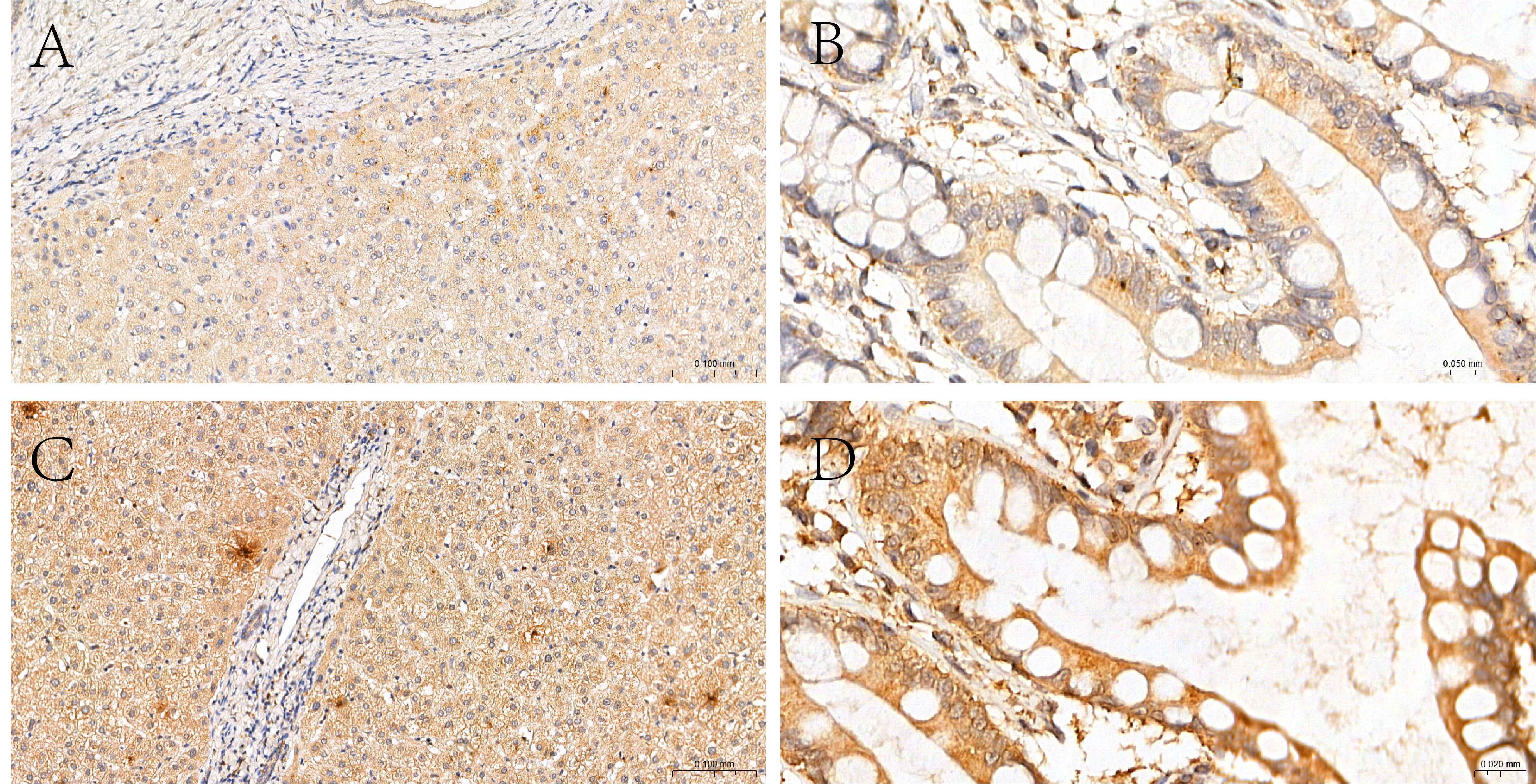

Supplement: Supplementary Figure 6 — Higher serum LEAP2 levels in patients with larger tumor burden. Each dot represents an individual level of LEAP2. [file Image6.jpeg]
