# Supplementary material for: Elevated blood levels of liver-expressed antimicrobial peptide 2 in patients with insulinoma and its expression in insulinomas
Source: Front Endocrinol (Lausanne). 2025 Dec 19;16:1685806. doi: 10.3389/fendo.2025.1685806 (PMC12757234; doi:10.3389/fendo.2025.1685806)
Supplement: Supplementary file 8 [file Table2.docx]

**Supplementary Table 2. Multivariable linear regression analysis of factors associated with serum LEAP2 levels.**

| **Variable** | **β (95% CI)** | **P value** |
| --- | --- | --- |
| Gender | 0.711 (-6.231-7.653) | 0.84 |
| Age | 0.061 (-0.191-0.313) | 0.63 |
| Insulin | 0.141 (0.020-0.262) | **0.02** |

Legend: Results from the multivariable linear regression model are presented as regression coefficient (β) with 95% confidence interval (CI). LEAP2, liver-enriched antimicrobial peptide 2.
