# Supplementary material for: Elevated blood levels of liver-expressed antimicrobial peptide 2 in patients with insulinoma and its expression in insulinomas
Source: Front Endocrinol (Lausanne). 2025 Dec 19;16:1685806. doi: 10.3389/fendo.2025.1685806 (PMC12757234; doi:10.3389/fendo.2025.1685806)
Supplement: Supplementary file 9 [file Table3.docx]

**Supplementary table 3** Correlation of LEAP2 expression in insulinoma with clinicopathological features

| **Clinicopathological characteristics** | **n** | **LEAP2 (+)** | **LEAP2 (-)** | **P value** |
| --- | --- | --- | --- | --- |
| Age | 26 | 49.9±12.4 | 44.0±21.6 | 0.440 |
| Gender | 26 |  |  |  |
| Male |  | 7(77.8%) | 2(22.2%) | 0.591 |
| Female |  | 15(88.2%) | 2(11.8%) |  |
| Primary tumor location | 25 |  |  |  |
| Pancreatic head and/or neck |  | 13(92.9%) | 1(7.1%) | 0.288 |
| Pancreatic body and/or tail |  | 8(72.7%) | 3(27.3%) |  |
| Tumor size | 26 |  |  |  |
| <2.5 cm |  | 19(82.6%) | 4(17.4%) | 1.000 |
| ≥2.5 cm |  | 2(100%) | 0 |  |
| Metastasis | 26 |  |  |  |
| No |  | 22(84.6%) | 4(15.4%) |  |
| Yes  Ki67  ≤2%  >2% | 11 | 0  7  1 | 0  3  0 | 0.782 |
| Grade | 19 |  |  |  |
| 1 |  | 14(82.4%) | 3(17.6%) | 1.000 |
| 2 |  | 2(100%) | 0 |  |
| Stage | 25 |  |  |  |
| I |  | 12(75.0%) | 4(25.0%) | 0.260 |
| II |  | 9(100%) | 0 |  |
